# The Australasian Triage Scale Level 5 Criteria may need to be revised

**Published:** 2017-01-14

**Authors:** Amir Mirhaghi, Mohsen Ebrahimi

**Affiliations:** 1 Evidence-Based Caring Research Center, Department of Medical-Surgical Nursing, School of Nursing and Midwifery, Mashhad University of Medical Sciences, Mashhad, Iran.; 2 Department of Emergency Medicine, Imam Reza Hospital, Mashhad University of Medical Sciences, Mashhad, Iran.

Australasian Triage Scale (ATS) is used to prioritize incoming patients in the emergency department (ED) according to patient acuity. It`s a five-level triage scale endorsed by the Australasian College for Emergency Medicine (ACEM). The ATS categories are defined by physiological predictors (airway, breathing, circulation, and disability) and maximum waiting time to treatment (1: immediate, 2: 10 minutes, 3: 30 minutes, 4: 60 minutes and 5: 120 minutes) (1). Triage scales should be valid and reliable to ensure safe practice and promote clinical applicability in ED (2). Ebrahimi et al. reported that the pooled coefficient for ATS is fair: 0.390 (95% CI 0.307–0.466) (3). 

I`d like to bring your attention to the fact that ATS has used the same criteria for level 4 and 5. Patients with normal Glasgow coma scale (GCS), patent airway and no respiratory distress and haemodynamic compromise may be allocated either to level 4 or 5 (1). Therefore, it has to be said that a source of confusion may exist in ATS level 4 and 5. 

Studies have also reported that ATS level 5 patients have not been recognized accurately and consistently by triage nurses (3, 4), despite the fact that ATS level 5 patient presentations are less urgent and usually easily distinguishable. 

However, these kinds of patients are not critically ill, their number is usually higher than patients of other categories and they may also be over triaged into upper categories by triage nurses and so urgent patients may encounter significant delay and harm. The reason may lay in adult physiological predictors (APP) in ATS that do not significantly differentiate between category 4 and 5 criteria (1). It`s also worth mentioning that contrary to emergency severity scale (ESI), ATS does not have a tendency to allocate patients into a specific category (5).

A recent study on validity and reliability of ATS has reported an overall inter-rater agreement of 0.40 using Fleiss’ kappa coefficient, representing a fair-to-good level of inter-rater agreement, as well as the fact that the lowest coefficient of reliability belongs to level 5 (0.47) (4).

Although, 65% of the participant responses to all triage scenarios were accurate, only 40% of triage decisions in level 5 has been accurate, representing the least value among triage categories (4). 

Therefore, fair consistency and low accuracy in category 5 raises serious questions that category 5 criteria may lack sufficient clarity, precision or accuracy.

Finally, since ATS needs to be modified periodically, like any other triage scale (6), it`s recommended that level 5 criteria should be revised to ensure safe practice in ED.

## References

[1] Aging DoHa (2009). Emergency Triage Education Kit: workbook.

[2] Esmailian M, Zamani M, Azadi F, Ghasemi F (2014). Inter-rater agreement of emergency nurses and physicians in Emergency Severity Index (ESI) triage. Emergency.

[3] Ebrahimi M, Heydari A, Mazlom R, Mirhaghi A (2015). The reliability of the Australasian Triage Scale: a meta-analysis. World J Emerg Med.

[4] Ekins K, Morphet J (2015). The accuracy and consistency of rural, remote and outpost triage nurse decision making in one Western Australia Country Health Service Region. Australasian Emergency Nursing Journal.

[5] Mirhaghi A, Kooshiar H, Esmaeili H, Ebrahimi M (2015). Outcomes for emergency severity index triage implementation in the emergency department. Journal of clinical and diagnostic research: JCDR.

[6] Considine J, Shaban RZ, FitzGerald GJ, Thomas S, Graham CA (2012). Triage and ATS: Collateral damage in the quest to improve ED performance. Australasian Emergency Nursing Journal.

